# Mitochondrial Fusion Is Increased by the Nuclear Coactivator PGC-1β

**DOI:** 10.1371/journal.pone.0003613

**Published:** 2008-10-31

**Authors:** Marc Liesa, Bárbara Borda-d'Água, Gema Medina-Gómez, Christopher J. Lelliott, José Carlos Paz, Manuel Rojo, Manuel Palacín, Antonio Vidal-Puig, Antonio Zorzano

**Affiliations:** 1 Institute for Research in Biomedicine (IRB Barcelona), Barcelona, Spain; 2 Departament de Bioquímica i Biologia Molecular, Facultat de Biologia, Universitat de Barcelona, Barcelona, Spain; 3 CIBER de Diabetes y Enfermedades Metabólicas Asociadas (CIBERDEM), Barcelona, Spain; 4 Institute of Metabolic Science, Metabolic Research Laboratories, University of Cambridge, Addenbrooke's Hospital, Cambridge, United Kingdom; 5 Department of Biosciences, AstraZeneca R&D, Mölndal, Sweden; 6 Institut de Biochimie et Génétique Cellulaires (IBGC) CNRS UMR5095, Université Victor Segalen, Bordeaux, France; Universität Heidelberg, Germany

## Abstract

**Background:**

There is no evidence to date on whether transcriptional regulators are able to shift the balance between mitochondrial fusion and fission events through selective control of gene expression.

**Methodology/Principal Findings:**

Here, we demonstrate that reduced mitochondrial size observed in knock-out mice for the transcriptional regulator PGC-1β is associated with a selective reduction in Mitofusin 2 (Mfn2) expression, a mitochondrial fusion protein. This decrease in Mfn2 is specific since expression of the remaining components of mitochondrial fusion and fission machinery were not affected. Furthermore, PGC-1β increases mitochondrial fusion and elongates mitochondrial tubules. This PGC-1β-induced elongation specifically requires Mfn2 as this process is absent in Mfn2-ablated cells. Finally, we show that PGC-1β increases Mfn2 promoter activity and transcription by coactivating the nuclear receptor Estrogen Related Receptor α (ERRα).

**Conclusions/Significance:**

Taken together, our data reveal a novel mechanism by which mammalian cells control mitochondrial fusion. In addition, we describe a novel role of PGC-1β in mitochondrial physiology, namely the control of mitochondrial fusion mainly through Mfn2.

## Introduction

Mitochondria are dynamic organelles whose morphology is regulated by fusion and fission processes. A growing body of evidence shows the relevance of these shaping processes in the control of mitochondrial activity and cell metabolism [1]–[7]. Several genes encoding mitochondrial fusion and fission proteins have been recently identified. Mammalian proteins involved in mitochondrial fission are Fission 1 homologue protein (Fis1) and Dynamin-related protein 1 (Drp1). Similarly, Mitofusin 1 (Mfn1), Mitofusin 2 (Mfn2) and Optic Atrophy gene 1 (OPA1) are proteins that participate in mitochondrial fusion in mammals [8]. However, there are no evidences to date that demonstrate the ability of an upstream or transcriptional regulator to shift the balance between mitochondrial fusion and fission events by selective regulation of these proteins.

Several reports provide evidence that Mfn2 protein elicits pleiotropic effects which may be involved in pathology. For instance, Mfn2 is mutated in Charcot Marie Tooth type 2A neuropathy [9] and, interestingly, some of these mutants cause selective defects in mitochondrial fusion [10], reduction in mitochondrial axonal transport [11] or defects in mitochondrial coupling leading to inefficient mitochondria [12]. Defective Mfn2 may also contribute to impaired mitochondrial function in the context of obesity and type 2 diabetes. This notion is supported by the observation that muscle Mfn2 expression is reduced in these patients [1], [13]. In addition, we have previously reported that Mfn2 can modulate mitochondrial activity through changes in the electron transport chain (ETC) and this modulation is independent of its role in mitochondrial morphology [1], [6].

Peroxisome proliferator-activated receptor γ coactivator-1 (PGC-1) α and β are important positive regulators of mitochondrial activity and biogenesis in mouse skeletal muscle [14]–[17]. Despite these similarities, PGC-1α and PGC-1β display low overall sequence identity, with the highest percentatges found in two particular domains (activation and RNA recognition domains, with identities of 40% and 50% respectively) [15]. Furthermore, key mitochondrial processes, such as organelle biogenesis and uncoupling, are differentially regulated by these homologues. For instance, in C2C12 muscle cells, PGC-1α but not PGC-1β, increases mitochondrial uncoupling, whereas PGC-1β causes a larger increase in mitochondrial volume than PGC-1α under the same conditions [18]. In addition, while PGC-1β expression in distinct tissues is unaffected by physiological processes characterized by increased energy expenditure, such as cold exposure (in brown adipose tissue), fasting (in liver) or exercise (in muscle), PGC-1α is highly regulated at the transcriptional level under similar physiological challenges [15]–[17]. These data suggest that PGC-1β likely controls basal mitochondrial biogenesis, whereas PGC-1α controls stimulated or regulated mitochondrial activity. In keeping with this view, PGC-1β expression is higher than PGC-1α expression in primary muscle cells under basal conditions [19].

The functional independency in mitochondrial physiology of these homologues is further illustrated by the phenotypes of PGC-1α and PGC-1β knockout (KO) mice. In both animal models, a general defect in the electron transport chain (ETC) system has been described, thereby demonstrating that PGC-1α does not fully compensate the effects of PGC-1β on mitochondria or vice versa [20]–[23]. Furthermore, several mitochondrial phenotypes described in the particular case of PGC-1β-ablated mice cannot be completely explained by impairment of the ETC system. For instance, muscle and liver from PGC-1β KO mouse show a reduction of mitochondrial volume without changes in mitochondria number [20], [23]. This decreased mitochondrial volume together with impaired ETC gene expression may explain the mitochondrial respiration defect found only in muscle strips and not in isolated mitochondria [20]. In keeping with these data, this reduction in mitochondrial size is absent in PGC-1α KO mouse under basal conditions, probably due to normal PGC-1β expression [21], [24]. Of note, despite all these differences, both genes show a diminished expression in the context of type 2 diabetes, suggesting an impairment of mitochondrial effects selectively regulated by each homologue in this disease [25], [26].

We previously reported that PGC-1α was able to induce Mfn2 transcription and that mitochondrial activity regulated by PGC-1α partly depends on correct Mfn2 expression. However, effects on mitochondrial fusion were not determined [27]. In the light of these results and the PGC-1β control of basal mitochondrial biogenesis, we aimed to determine whether PGC-1β was able to control Mfn2 transcription and, therefore, if the mitochondrial dynamics balance could be modulated by transcriptional regulation. Here, we address these questions by determining the role of PGC-1β in the control of mitochondrial fusion using a multi-approach strategy that combines Mfn2 KO cells, PGC-1β-overexpressing muscle cells and PGC-1β-ablated mice.

## Results

### PGC-1β induces Mfn2 transcription through ERRα coactivation

**Figure 1:**
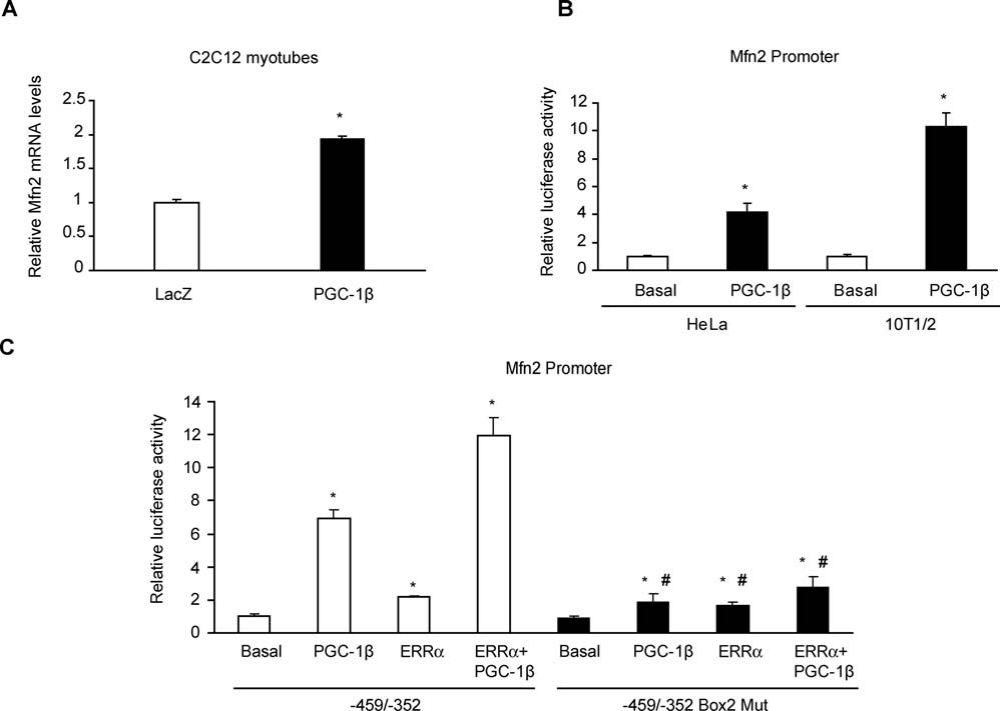
PGC-1β increases Mfn2 transcription through nuclear receptor ERRα. (A) Total RNA was obtained from C2C12 myotubes transduced with LacZ (white bars) or PGC-1β (black bars) adenovirus at a MOI 100 from 3 independent experiments in duplicate. Mfn2 and β-Actin mRNA levels were measured by real-time PCR. Results are mean±SEM and are expressed relative to β-Actin values, *, statistical difference at p<0.001. (B) Transcriptional activity of −1982/+45 human Mfn2 promoter was determined by luciferase activity (corrected by renilla activity) in 10T1/2 and HeLa cells. Results are mean±SEM from 3 independent experiments performed in triplicate. Cells were transfected with 200 ng of an irrelevant vector (Basal, white bars) or PGC-1β expression vector (PGC-1β, black bars) and expressed relative to the basal group; * statistical difference at p<0.05. (C) Luciferase activity (corrected by renilla) from 10T1/2 cells transfected with a −459/−352 Mfn2 promoter fragment containing a mutation that disrupted box 2 (−459/−352 Box 2 Mut, black bars) or with the wild-type fragment (−459/−352, white bars), together with an irrelevant vector (Basal), ERRα, 200 ng of PGC-1β or ERRα+PGC-1β expression plasmids. Mean±SEM values from 3 independent transfection experiments performed in triplicate are shown. *, statistical difference compared with basal groups, p<0.05. #, statistical difference compared with the wild-type promoter fragment activity, p<0.05.

Our initial aim was to determine whether PGC-1β regulates the expression of Mfn2 in C2C12 muscle cells. Differentiated C2C12 cells show low levels of PGC-1β mRNA as assessed by Northern blot [18] and by real-time PCR (data not shown). To this end, C2C12 myotubes were transduced either with a mouse PGC-1β adenovirus or with a control LacZ adenovirus. Mfn2 mRNA levels doubled in PGC-1β-expressing muscle cells compared to control transduced myotubes (Fig. 1A). To demonstrate that PGC-1β directly increases Mfn2 transcription, we transfected 10T1/2 mouse fibroblasts or HeLa cells with a construct containing a 2-kb fragment (−1982/+45) of the Mfn2 promoter fused to a luciferase reporter gene, together with an irrelevant vector (Basal) or mouse PGC-1β expression vector (PGC-1β). PGC-1β markedly enhanced Mfn2 promoter activity (10.3±0.9- and 4.2±0.6-fold values over basal Mfn2 promoter activity in 10T1/2 and HeLa respectively, Fig. 1B). In a previous study, using electrophoretic mobility shift and chromatin immunoprecipitation assays, we showed that ERRα binds to Mfn2 promoter between nucleotides −459/−396 [27]. This DNA region contains three putative boxes with the capacity to bind nuclear receptors, where box 2 is critical for Mfn2 promoter response to PGC-1α coactivation of ERRα [27]. On the basis of these observations, we determined whether PGC-1β coactivated ERRα through box 2 in a similar way as PGC-1α. We transfected 10T1/2 cells with a construct containing a −459/−352 Mfn2 promoter fragment fused to a luciferase reporter gene or with a mutated version of the same fragment that disrupted box 2 [27]. We observed a marked coactivation of ERRα by PGC-1β in the −459/−352 Mfn2 promoter fragment (Fig. 1C). This effect was completely blunted when box 2 was disrupted (11.9±1.09 vs. 2.8±0.6-fold over basal promoter activity, p = 0.001, Fig. 1C). Furthermore, cancellation of box 2 markedly reduced the activation driven by PGC-1β (7.0±0.5 vs. 1.9±0.5, p = 0.001) or by ERRα (2.2±0.1 vs. 1.6±0.2, p = 0.04), although residual activation was still present. Similar results were obtained when the 2- kb Mfn2 promoter was cotransfected with ERRα or PGC-1β (Figure S1).

### PGC-1β causes a large induction of Mfn2 protein levels

**Figure 2:**
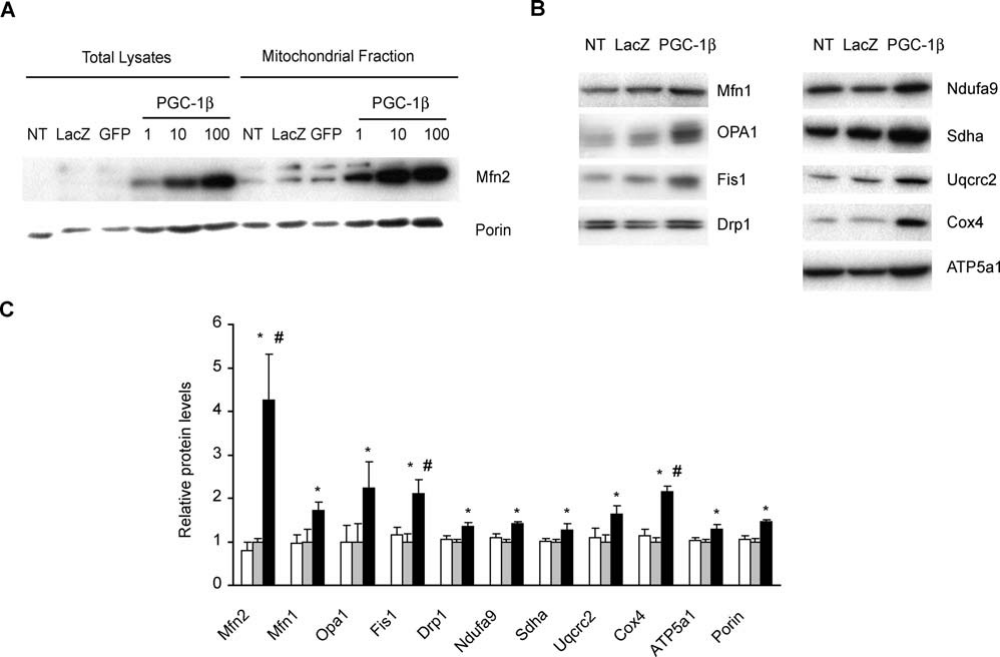
Effect of PGC-1β on the expression of Mfn2, other proteins involved in mitochondrial dynamics, Porin and ETC subunits. Total lysates and mitochondrial fractions were obtained from C2C12 myotubes 48 h after adenoviral transduction, and the expression of proteins was analyzed by Western blot (40 µg). Representative films of a single experiment from four independent differentiation and transduction experiments are shown. (A) Total lysates and mitochondrial fractions of non-transduced myotubes (NT), transduced with LacZ or GFP control adenovirus at a MOI 100 (control adenovirus at a MOI 1 and 10 displayed no changes, data not shown) or with PGC-1β adenovirus at a MOI 1, 10 or 100 were probed with anti-Mfn2 and Porin antibodies. (B) Specific antibodies were used to detect mitochondrial dynamics proteins (Mfn1,OPA1, Drp1 and Fis1) and single subunits from each complex of the ETC (complex I, Ndufa9; complex II, Sdha; complex III, Uqcrc2; complex IV, Cox4 and complex V, ATP5a1) in mitochondrial fractions of non-transduced (NT) or transduced C2C12 myotubes at a MOI 100 with LacZ or PGC-1β adenovirus. (C) Quantification analysis of protein levels of mitochondrial dynamics components and the ETC subunits from complexes I to V detected by Western blot. Mean±SEM of Non- (white bars) , LacZ- (grey bars) or PGC-1β- (black bars) transduced C2C12 myotubes from n = 4 independent differentiation and transduction (MOI 100) experiments. *, statistical difference compared to LacZ transduction, p<0.05. #, statistical difference compared to Porin induction, p<0.05.

To determine whether PGC-1β-mediated Mfn2 transcription leads to enhanced Mfn2 protein expression, we transduced C2C12 myotubes with PGC-1β or two distinct control adenoviruses at a multiplicity of infection (MOI) of 1, 10 or 100. Total protein extracts and mitochondrial-enriched fractions were obtained and analyzed by Western blot. PGC-1β induced Mfn2 in muscle cells (Fig. 2A) and a direct relationship between PGC-1β adenoviral dose and Mfn2 protein induction was detected (Fig. 2A). PGC-1β also increased the cellular content of the constitutive mitochondrial protein Porin (Fig. 2A), used as a measure of mitochondrial mass. Densitometric quantification of Porin induction at MOI 100 showed a 1.8±0.2-fold increase in total lysates (p = 0.01, Figure S2) and 1.45±0.003-fold in mitochondrial-enriched fractions (p = 0.002, Fig. 2C). Porin induction values in total lysates are consistent with the increase in mitochondrial mass volume reported in PGC-1β-overexpressing C2C12 muscle cells [18].

**Figure 3:**
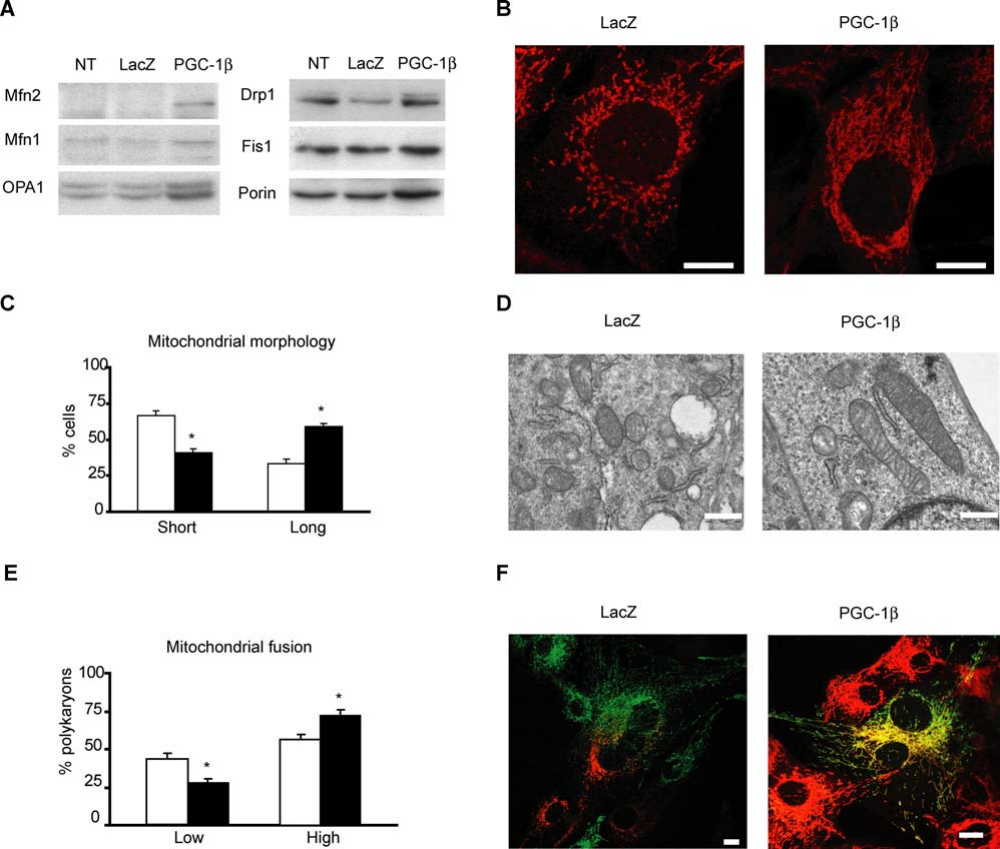
PGC-1β increases the rate of mitochondrial fusion and changes mitochondrial morphology. (A) Representative Western blot of three independent experiments of C2C12 myoblasts transduced with LacZ or PGC-1β adenovirus at a MOI 200 during 48 h. 40 µg of protein from lysates were loaded and Mfn2, Mfn1, Opa1, Drp1, Fis1 and Porin were detected with specific antibodies. (B) Representative images from a duplicate of one of the same three independent transduction experiments performed in panel (A). Mitochondria were labelled with anti-Cox1 antibody detected with a secondary antibody conjugated to Alexa 594. Scale bar, 10 µm. (C) C2C12 myoblasts quantification and classification attending to mitochondrial tubule length (150–200 cells counted per transduction). Myoblasts with a mean mitochondrial tubule length <4 µm were considered “Short” and >4 µm were considered “Long”. Results are mean±SEM and expressed as percentage of total C2C12 myoblasts counted from three independent transduction experiments with LacZ (white bars) or PGC-1β (black bars) at MOI of 200. *, statistical difference at p<0.01. (D) Electron microscopy images of C2C12 myoblasts transduced with LacZ or PGC-1β adenovirus as in panel (A) were taken at 40.000x magnification . Scale bar 500 nm. (E) 4 hours after PEG, polykaryons of C2C12 myoblasts transduced as in (A) were counted attending to mtGFP (mitochondrial matrix- targeted green fluorescent protein) and mtRFP (mitochondrial matrix-targeted red fluorescent protein) mixing (detected as yellow mitochondria). Polykaryons classified as “Low” displayed <50% of mixed mtGFP and mtRFP (yellow mitochondria). Polykaryons classified as “High” displayed ≥50% of yellow mitochondria. Graphs show mean±SEM of percentage of total polykaryons counted from three independent cell fusion experiments and transduction experiments with LacZ (white bars) or PGC-1β (black bars) at MOI of 200. *, statistical difference at p = 0.02. (F) Representative images from one of three independent transduction and polyethylene glycol-mediated (PEG) cell fusion experiments. C2C12 myoblasts stably expressing mitochondrial matrix-targeted green fluorescent protein (mtGFP) or red fluorescent protein (mtRFP) were transduced with LacZ or PGC-1β adenovirus as in panel (A). Polykaryons were fixed 4 h after 30”–40” PEG treatment. During these 4 h, cells were incubated in growth medium with 100 µg/ml of cycloheximide to inhibit *de novo* synthesis of mtGFP and mtRFP. Degree of fusion is directly proportional to mixing of mtGFP and mtRFP (yellow mitochondria). Scale bar, 10 µm.

On the basis of the effects of PGC-1β on Mfn2 and Porin expression, we also analyzed whether this coactivator regulates the expression of other proteins involved in mitochondrial dynamics and in the ETC system. PGC-1β-transduced myotubes induced the expression of Mfn1, OPA1, Drp1 and Fis1 (Fig. 2B) in mitochondrial-enriched extracts (1.7-, 2.2-, 1.4-, or 2.1-fold values over basal values, respectively) to a level similar to that detected for Porin (1.45-fold stimulation, Fig. 2C). Mfn2 displayed a significantly higher increase in expression compared to Porin abundance in mitochondrial-enriched extracts (4.3-fold induction, Fig. 2C). When data were expressed as protein levels relative to Porin expression, we only detected significant stimulation of Mfn2 and Fis1 in response to PGC-1β over-expression (Figure S3). Similar results were obtained in total lysates (data not shown). This superior induction of Mfn2 protein was also detected in C2C12 myoblasts (Fig. 3A).

We also analyzed several subunits of the ETC system in mitochondrial-enriched fractions from C2C12 myotubes transduced at MOI 100 (Fig. 2B,C). All the subunits of complexes I, II, III, IV and V studied were induced in response to PGC-1β (Fig. 2B,C), and the extent of induction was similar to that detected in Porin levels (ranging from 1.3- to 2.1-fold increase).

### PGC-1β changes mitochondrial morphology and increases the rate of mitochondrial fusion

As PGC-1β induces Mfn2 expression, we next determined whether it was linked to changes in mitochondrial morphology. To this end, we immunofluorescently labelled mitochondria from C2C12 myoblasts transduced with PGC-1β or LacZ adenovirus. PGC-1β caused an increase in the length of mitochondrial tubules (Fig. 3B) in most myoblasts (59% vs. 33%, in PGC-1β and in control cells, respectively, p = 0.003) (Fig. 3C). This increased mitochondrial size induced by PGC-1β overexpression was also observed by transmission electron microscopy, showing normal cristae morphology (Fig. 3D). To demonstrate that this increase in mitochondrial length was linked to an increase in mitochondrial fusion, we performed a polyethylene glycol-(PEG) mediated cell fusion assay by using two distinct C2C12 lines stably expressing mitochondrial matrix-targeted GFP (mtGFP) or red fluorescent protein (mtRFP) [28]. Four hours after PEG and in the presence of cycloheximide, a significantly higher percentage (73% vs. 55% in PGC-1β and control cells, respectively, p = 0.01) (Fig. 3E) of polykaryons from C2C12 transduced with PGC-1β showed a higher level of mitochondrial matrix content mixing than control LacZ polykaryons (that is mtGFP and mtRFP exchange due to mitochondrial fusion and displayed as yellow mitochondria) (Fig. 3F). This higher mtGFP and mtRFP mixing indicated an increase in the rate of mitochondrial fusion induced by overexpression of PGC-1β. In agreement with previous data [18], the overexpression of PGC-1β in C2C12 myoblasts under these conditions also increased a ∼50% the mitochondrial membrane potential values measured using the fluorescent probe JC-1 (data not shown).

### Mfn2 is required for PGC-1β-induced changes in mitochondrial morphology

**Figure 4:**
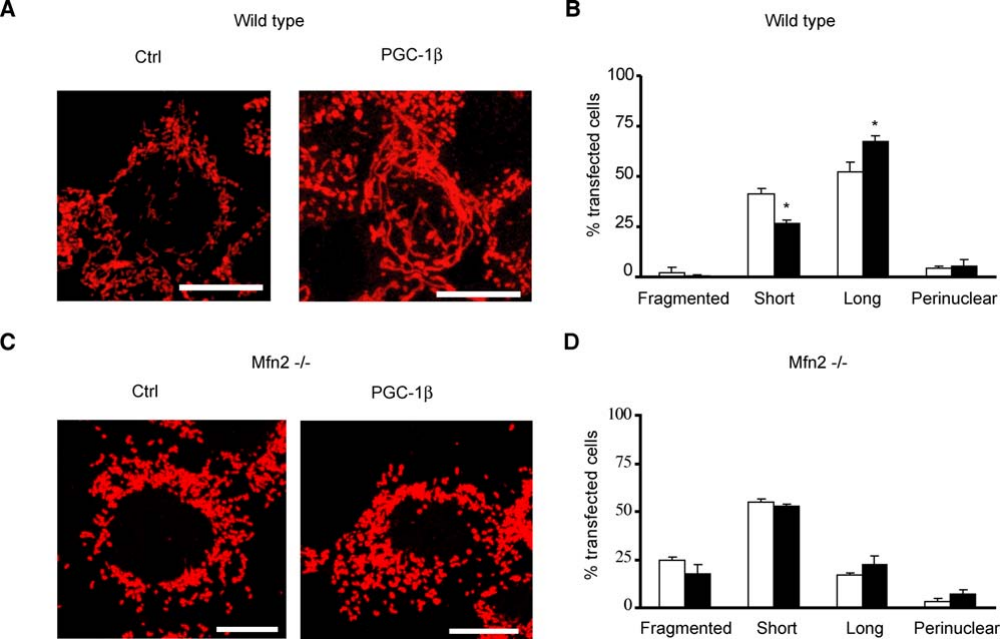
Mfn2 is required for PGC-1β changes in mitochondrial morphology. (A) Representative immunocytochemistry images (performed as in Fig. 3B) from one of three independent cotransfection experiments with an empty vector (Control) or PGC-1β- encoding vector (PGC-1β) and GFP (ratio GFP:PGC-1β 1:10) of wild-type mouse embryonic fibroblasts (MEFs). Transfected cells were visualized by GFP fluorescence (data not shown). Scale bar, 10 µm. (B) Wild type mouse embryonic fibroblasts (MEF) quantification and classification attending to mitochondrial tubule length (50-75 transfected cells counted per independent experiment). Wild type MEFs with a mean mitochondrial tubule length <4 µm were considered “Short” and >4 µm were considered “Long”. “Perinuclear” MEFs displayed most mitochondria surrounding the nucleus (see Figure S4) and almost no “Fragmented” cells (with tiny spherical mitochondria, see Figure S4) were observed in transfected wild type MEFs. Results are mean±SEM and expressed as percentage of total transfected wild type MEFs counted from three independent cotransfection experiments with an empty vector (white bars) or PGC-1β (black bars) with GFP (empty vector/PGC-1β : GFP ratio 10:1). Transfected cells were observed by GFP fluorescence (data not shown). *, statistical difference at p<0.02. (C) Representative immunocytochemistry images (performed as in Fig. 3B) from one of three independent cotransfection experiments with an empty vector (Control) or PGC-1β- encoding vector (PGC-1β) and GFP (ratio GFP:PGC-1β 1:10) of Mfn2 −/−mouse embryonic fibroblasts (MEFs). Transfected cells were visualized by GFP fluorescence (data not shown). Scale bar, 10 µm. (D) Mfn2 −/− mouse embryonic fibroblasts (MEF) quantification and classification attending to mitochondrial tubule length (100–250 transfected cells counted per independent experiment). Mfn2 −/− MEFs with a mean mitochondrial tubule length <4 µm were considered “Short” and >4 µm were considered “Long”. “Perinuclear” MEFs displayed most mitochondria surrounding the nucleus (see Figure S4) and a significant percentage of “Fragmented” cells (with tiny spherical mitochondria, see Figure S4) were observed in transfected Mfn2 −/− MEFs. Results are mean±SEM and expressed as percentage of total transfected Mfn2 −/− MEFs counted from three independent cotransfection experiments with an empty vector (white bars) or PGC-1β (black bars) with GFP (empty vector/PGC-1β : GFP ratio 10:1). Transfected cells were observed by GFP fluorescence (data not shown).

To study whether the change in mitochondrial morphology induced by PGC-1β was mediated mainly through Mfn2 activity, we used mouse embryonic fibroblasts (MEFs) from wild-type and from Mfn2 or Mfn1 KO mice [29]. Overexpression of PGC-1β led to an increase in mitochondrial length of wild-type (Fig. 4A,B) and Mfn1 KO MEFs (data not shown), similarly as observed in C2C12 myoblasts (Fig. 3B,C). Importantly, PGC-1β gain-of- function was unable to promote mitochondrial elongation in Mfn2 KO MEFs (Fig. 4C,D), in conditions in which ETC subunits Cox4 and Uqcrc2 were increased 1.6- and 1.5-fold respectively (data not shown). These data demonstrate the requirement of Mfn2 expression for PGC-1β-mediated changes in mitochondrial morphology.

### PGC-1β KO mice show Mfn2 repression in skeletal muscle and miocardium

**Figure 5:**
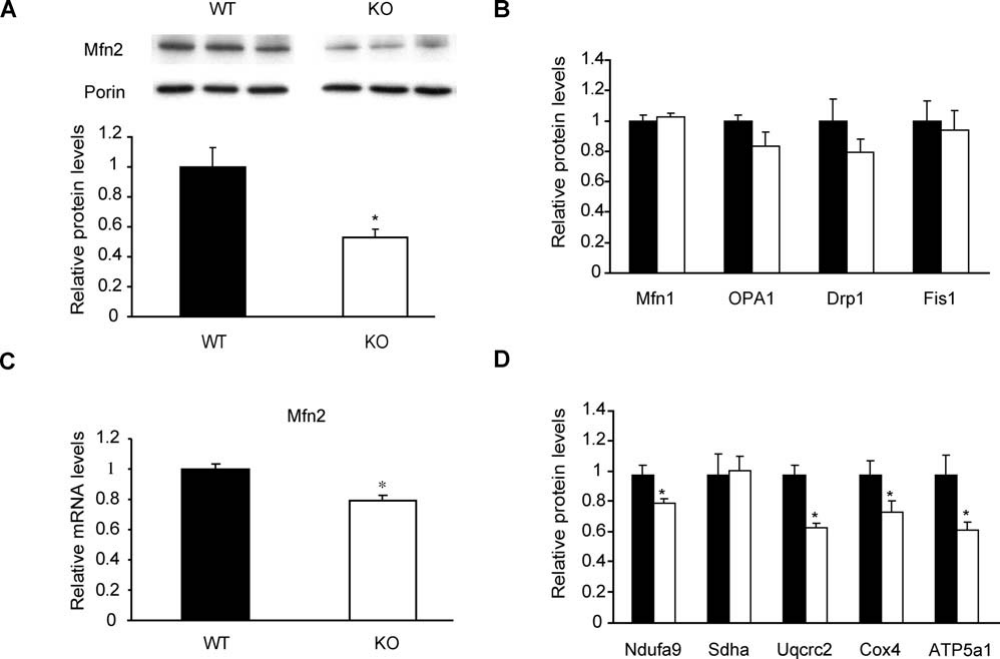
PGC-1β KO mice show a specific decrease in Mfn2 expression in skeletal muscle. Gastrocnemius muscles from 4- or 8-month-old wild-type (WT, black bars) or PGC-1β KO male mice (KO, white bars) were used to obtain total RNA and protein. (A) Representative image from a Western blot with specific detection of Mfn2 and Porin in muscle lysates. Graph represents mean±SEM of Mfn2 levels relative to Porin values (n = 6 mice per group). *, statistical difference at p<0.01. (B) Abundance of proteins involved in mitochondrial dynamics (Mfn1, OPA1, Drp1 and Fis1) in total lysates. Graph represents mean±SEM of protein levels relative to Porin values (n = 6 mice per group). (C) Real-time PCR analysis of Mfn2 from WT and KO mice. Graph represents mean±SEM and data are expressed as values of Mfn2 relative to 36B4 mRNA levels (n = 8 mice per group). *, statistical difference at p<0.01. (D) Abundance of the ETC subunits in mitochondrial fractions (n = 6 mice per group). Subunits of complexes I to V of the ETC were detected with specific antibodies. Graph represents mean±SEM and data are expressed as values relative to Porin expression. *, statistical difference at p<0.05.

We next studied the effects of in vivo ablation of PGC-1β on Mfn2 expression in gastrocnemius muscles. Mfn2 protein levels were reduced by approximately 50% in KO mice (Fig. 5A). Reduction of Mfn2 levels was relatively specific as indicated by the absence of major changes in proteins involved in mitochondrial dynamics, i.e., Mfn1, OPA1, Drp1 and Fis1 (Fig. 5B). Similar results were obtained using mitochondrial fractions (data not shown). Reduced Mfn2 protein expression paralleled lower levels of Mfn2 mRNA (Fig. 5C).

Soleus muscle from PGC-1β KO mice also shows a decrease in complex IV (subunits Cox4 and Cox5b) mRNA levels [20]. This observation led us to study whether the protein levels of Cox4 and other subunits of complexes I, II, III and V were also altered in gastrocnemius muscle of this mouse model. These mice displayed approximately a ∼40% reduction in complex V (ATP5a1) and III (Uqcrc2) (Fig. 5D). Complex IV (Cox4) showed a ∼25% reduction and complex I (Ndufa9) a ∼20% decrease while no changes were observed in complex II protein levels (Sdha) (Fig. 5D).

The heart is one of the organs with the highest expression of PGC-1β [16]. Interestingly, heart lysates from KO mice displayed a clear reduction in Mfn2 protein levels (WT 1.00±0.06, KO 0.57±0.09, p = 0.0015; data not shown), suggesting the impairment of this regulatory pathway also in cardiac muscle.

### Hepatic Mfn2 levels are reduced in PGC-1β KO mice

**Figure 6:**
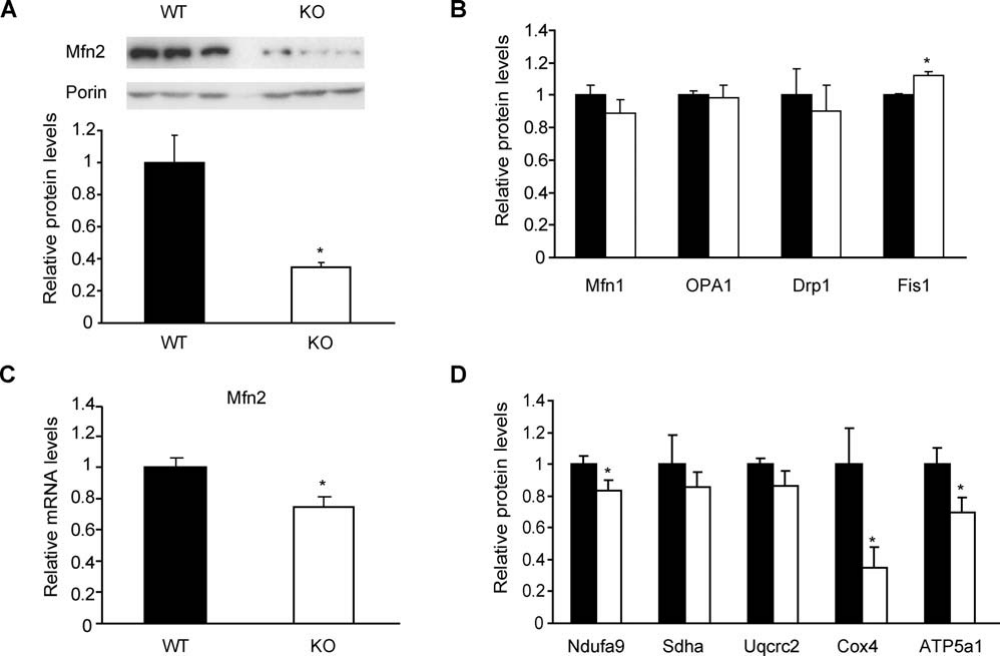
Hepatic Mfn2 levels are diminished in PGC-1β KO mice. Livers from 4- or 8- month-old wild-type (WT, black bars) and PGC-1β KO male mice (KO, white bars) were used to obtain total RNA and protein. (A) Representative image from a Western blot with specific detection of Mfn2 and Porin in liver lysates from WT or KO mice. Graph represents mean±SEM of Mfn2 levels related to Porin (n = 6 mice per group). *, statistical difference at p<0.01. (B) Abundance of proteins involved in mitochondrial dynamics in total lysates (Mfn1, OPA1, Drp1 and Fis1). Graph represents mean±SEM of protein levels relative to Porin values (n = 6 mice per group). (C) Real-time PCR analysis of Mfn2 from WT and KO mice. Graph represents mean±SEM and data are expressed as values of Mfn2 relative to 36B4 mRNA levels (n = 8 mice per group). *, statistical difference at p<0.01. (D) Abundance of the ETC subunits in mitochondrial fractions (n = 6 mice per group). Single subunits of complexes I to V of the ETC were detected with specific antibodies. Graph represents mean±SEM and data are expressed as values relative to Porin expression. *, statistical difference at p<0.05.

Liver is a crucial metabolic organ and shows high mitochondrial activity. PGC-1β KO mice also show reduced hepatic ETC gene expression [20]. On the basis of these observations, we next examined whether hepatic Mfn2 expression was altered in PGC-1β KO mice. Hepatic lysates from PGC-1β KO mice were obtained and analyzed by Western blot. Mfn2 protein levels were reduced by 65% in KO livers compared to the control group (Fig. 6A). This decrease was specific since among other proteins involved in mitochondrial dynamics only a modest 12% increase in Fis1 levels was detected (Fig. 6B). Similar results were obtained in mitochondrial fractions (data not shown). Mfn2 mRNA levels were also reduced by 25% in livers from PGC-1β KO mice (Fig. 6C).

Hepatic PGC-1β expression is markedly induced in mice fed a high fat diet, which is, to our knowledge, the only situation reported to induce PGC-1β mRNA levels in adult tissues. Therefore, we analyzed hepatic Mfn2 expression in wild-type and PGC-1β KO mice fed a high fat diet during 6 months. We observed a higher reduction in Mfn2 mRNA levels in livers from PGC-1β KO mice fed a high fat diet (by 45%, data not shown) compared to PGC-1β KO mice fed a chow diet (by 25 %). This was specific to Mfn2 and no changes in Mfn1 expression were detected under normal or high fat diet conditions (data not shown).

The expression of some ETC subunits was examined in liver mitochondrial fractions of PGC-1β KO mice (Fig. 6D). Cox4 levels were reduced by 65%. Subunits of complexes I and V displayed moderate reductions (∼20% and ∼30%, respectively), while those of complexes II and III remained unaltered (Fig. 6D).

## Discussion

In the present study we demonstrate that mitochondrial dynamics balance can be shifted towards fusion by transcriptional regulation. More specifically, we show that PGC-1β is a regulator of mitochondrial fusion through its effects of selectively promoting Mfn2 expression upon coactivation of ERRα. This new role of PGC-1β in mitochondrial physiology has been demonstrated using both in vitro (muscle cells) and in vivo (PGC-1β-ablated mice) approaches. Firstly, we have shown that PGC-1β overexpression in muscle cells regulates mitochondrial dynamics through a mechanism that involves the preferential induction of Mfn2 expression, among other mitochondrial dynamics effectors such as Mfn1, OPA1, Drp1 and Fis1. Furthermore, PGC-1β gain-of-function results in an elongation of mitochondrial tubules, which is linked to an increased mitochondrial fusion. Importantly, the effects of PGC-1β promoting mitochondrial elongation are not observed in Mfn2-ablated cells, thereby demonstrating that Mfn2 activity is essential for PGC-1β-mediated changes in mitochondrial dynamics.

The relevance of these observations is confirmed by our in vivo data showing that PGC-1β KO mice have a specific reduction of Mfn2 expression in skeletal muscle whereas Mfn1, OPA1, Drp1 and Fis1 levels are maintained. In fact, this preferential regulation of Mfn2 protein levels could explain the reduction in mitochondrial volume, without changes in mitochondria number, observed in liver and muscle from PGC-1β loss-of-function mice [20], [23]. We propose that Mfn2-impaired function in PGC-1β KO mice prevents a normal rate of mitochondrial fusion while physiological mitochondrial fission events remain unaffected, thereby resulting in smaller mitochondria as previously reported for Mfn2 KO or antisense cells [1], [30].

Several lines of evidence demonstrate the relevance of maintaining a proper balance between fusion and fission processes for the specific mitochondrial activity required by distinct cell types [1]–[6]. In this regard, PGC-1β expression is higher in tissues with marked mitochondrial activity (such as liver, muscle and heart), a similar pattern to that reported for Mfn2 [1], [16]. In addition, we have found that PGC-1β regulates Mfn2 expression in liver, skeletal muscle and heart, as PGC-1β KO mice show a marked decrease in Mfn2 in those tissues. The correlational data mentioned before together with the observations obtained in skeletal muscle, heart and liver from PGC-1β KO mice indicate that PGC-1β controls basal expression of Mfn2 in these tissues.

On the basis of these observations, we propose that increased mitochondrial fusion caused by PGC-1β-induced Mfn2 contributes to a fully optimal mitochondrial activity in muscle and liver tissues, which may be defective in skeletal muscle of type 2 diabetic patients. Furthermore, we propose that PGC-1β controls basal mitochondrial fusion in skeletal muscle, whereas PGC-1α would probably control mitochondrial fusion in high energy expenditure situations, such as cold-exposure or exercise, conditions which largely induce Mfn2 expression [27]. This is supported by the fact that PGC-1β expression is higher than PGC-1α expression in skeletal muscle under basal conditions and by the lack of a decrease in mitochondrial size in PGC-1α KO mice [19], [24]. In keeping with this proposal, our data permit to explain the observation of a defective mitochondrial respiration found in muscle strips but not in isolated mitochondria from PGC-1β KO mice under basal conditions [20]. In isolated mitochondria, the integrity of the mitochondrial network and dynamics inside the cell is not maintained and therefore defects in mitochondrial activity secondary to abnormal mitochondrial dynamics are not detectable.

Here, we have also examined the mechanism by which PGC-1β induces Mfn2 transcription, and have identified ERRα as the key transcription factor coactivated by PGC-1β on the Mfn2 promoter. The effect of ERRα and PGC-1β occurred at the level of box 2 (located in the −459/−352 promoter region), although PGC-1β also displayed additional stimulatory actions on the Mfn2 promoter. The same box 2 region is the major component required for PGC-1α activation of the Mfn2 promoter [27]. On the basis of these data, we conclude that PGC-1α and PGC-1β display a common mechanism of activation of the Mfn2 promoter, through nuclear receptor ERRα. However, despite using the same response elements, decreased expression of Mfn2 induced by genetic ablation of PGC-1β in mice cannot be counteracted by PGC-1α action, as shown in liver, heart and skeletal muscle of PGC-1β KO mice, in which Mfn2 expression is decreased, probably due to the low expression of PGC-1α under basal conditions.

In summary, we provide evidence that mitochondrial dynamics balance is selectively controlled by a transcriptional regulator, unravelling an upstream mediator of mitochondrial fusion. Furthermore, we also provide evidence of a novel role of PGC-1β in mitochondrial physiology. Given the crosstalk between mitochondrial activity and dynamics, together with reduced Mfn2 and PGC-1β expression in type 2 diabetes, we conclude that the pathway reported here is not only relevant for the thorough explanation of mitochondrial dynamics regulation and the overall mitochondrial effects of PGC-1β, but also may provide the basis for the correct understanding of the alterations of mitochondrial metabolism associated with type 2 diabetes.

## Materials and Methods

### Animal Care and tissue collection

All animal protocols were approved as previously published [20]. Mice were cared for according to the Guiding Principles for Research Involving Animals and Human Beings. Tissue collection was performed as previously published [20].

### Cell culture, transfections, luciferase assays and adenoviral transductions

10 T1/2 , HeLa , HEK293A and C2C12 cell lines were grown as reported [27].

10 T1/2 fibroblasts and HeLa cells were transfected in 24-well plates at 40% confluence using Lipofectamine 2000 (Invitrogen) and harvested 36 h after. Transfected DNA plasmids were 1.04 µg/well and ratios/well were 75 ng to 200 ng of PGC-1β, 200 ng of ERRα, 600 ng of reporter, 40 ng of Renilla and up to 1.04 µg/well of pcDNA 3.1 as an irrelevant vector. 10 µl of total lysates from 10 T1/2 or HeLa cells were analysed using Dual-Glo™ Luciferase Assay System from Promega following the manufacturer's instructions.

Mouse embryonic fibroblasts (MEFs) from wild-type, Mfn1 KO and Mfn2 KO mice [29] were seeded in coverslips 24 h before cotransfection with an empty vector or with a plasmid encoding mouse PGC-1β and GFP (empty vector/PGC-1β:GFP ratio 10:1) in 6-well plates at 40% confluence using Transfectin reagent (1 µg DNA : 3 µl reagent) (BioRad). Transfection efficiencies for wild-type MEFs were ∼5–15% and for Mfn2 −/− MEFs were ∼40–60% (observed by GFP fluorescence). MEFs were fixed 48 h after transfection.

C2C12 differentiated myotubes were obtained 3 days after adding differentiation medium to 90% confluent cells (5% Horse Serum from Gibco, P/S and 25 mM HEPES). Myotubes were transduced with a LacZ- or a mouse PGC-1β-encoding adenovirus at a multiplicity of infection (MOI) of 1, 10 or 100 during 24 h and processed 2 days after transduction. Myoblasts were transduced at a MOI 200, as they are less sensitive to adenoviral transduction compared to myotubes [31]. Myotubes transduced at a MOI 100 and myoblasts at a MOI 200 displayed 99% of transduced cells (data not shown).

### Plasmids and adenoviruses

Constructs and adenovirus were previously described [27], [32]. Adenovirus titer was determined in HEK293A cells as previously described [6].

### Real Time PCR analysis

Taqman Probes 20X (Applied Biosystems) for Mfn2, PGC-1β and 3B64 were used. β-Actin primers, RNA isolation, cDNA synthesis and PCR conditions were as described in [27], [32] . Relative expression levels were calculated by ΔCt and the standard curve method [27], [32].

### Protein extracts and subcellular fractionation

Total lysates and mitochondria from C2C12 myoblasts and myotubes, and skeletal muscle, liver and heart from PGC-1β KO mice were isolated by differential centrifugation as previously described [6]. Protein obtained was quantified by the BCA method (Pierce).

### Western blot and antibodies used

SDS-PAGE, loading controls and densitometric analysis were performed as previously described [6]. Images were processed with Adobe Photoshop CS adjusting levels to reduce background.

The monoclonal antibodies against the ETC system subunits and Porin were described in our previous study [6]. OPA1 and Drp1 antibodies were purchased from BD Biosciences Pharmingen (San Diego, California, USA) and used at a 1/1000 dilution. Polyclonal antibodies generated in rabbit: serum raised against Mfn2 [6] was affinity purified by Sulfo-Link Kit (Pierce, Rockford, Illinois, USA), conjugating the immunogenic peptide (Eurogentec, Liege, Belgium) with an N-terminal cysteine (Nter C-LGPKNSRRALMGYNDQVQRP Cter) to a column and used at dilution 1/500. Mfn1 antibody was from Legros et al. [28] and Fis1 antibody from BioVision (Mountain View, California, USA) (1/500). All antibodies were diluted in PBS 5% dry non-fat milk and incubated overnight at 4°C with orbital agitation. Detection of primary antibodies was performed as previously described [6].

### Transmission electron microscopy

Cells were fixed with PFA 2%/Glutatraldehyde 0.125% in the plate and scrapped gently after 60' of fixation at room temperature. The rest of the protocol was performed as previously reported [6]. Images were taken at 40000x magnification in the electron microscopy platform, Serveis Científico-Tècnics (SCT), Universtitat de Barcelona.

### Immunocytochemistry and microscopy

Immunocytochemistry of C2C12 myoblasts and MEFs was performed as previously described, using the fluorochrome Texas red (conjugated to an anti-mouse secondary antibody from Molecular probes) [6] and coverslips were mounted in Mowiol (Calbiochem). Images were taken at room temperature with a Leica laser scanning confocal microscope SP2 (laser excitation 594 nm) using a 63X oil immersion objective (1.4). Images were magnified 5 times with Leica Lite ® software.

Images without magnification (with 30–40 cells per field) were used to count 150–200 cells, which were classified in two groups. Cells with a mean mitochondrial length >4 µm were considered to have elongated mitochondria (Long), the rest were considered short (Short). Mean mitochondrial length was determined (in images magnified 5 times) by measuring the length of 40–60 mitochondrial filaments per cell with Leica Lite ® software.

In the particular case of MEFs, transfected cells were counted (GFP-positive, laser 488 nm excitation, from 60 to 200 cells counted per independent experiment and transfection). In Mfn1 and Mfn2 −/−, we observed two additional cell populations, on the basis of their mitochondrial morphology (see Figure S4), which we named “fragmented”, given that most mitochondria from these cells were spherical (similar percentages as previously reported, [3]) , and “perinuclear”, for which almost all mitochondria located surrounding the nucleus.

### C2C12 myoblasts stably expressing mtGFP and mtRFP

Mitochondrial matrix-targeted GFP and RFP [28] were PCR amplified (with 5′ BamHI and 3′ EcoRI primers) and cloned into pGEM-T easy vector (Promega). Inserts, cloned into pGEM-T, were obtained by EcoRI-BamHI (New England Biolabs) digestion and were ligated (New England Biolabs) into BamHI EcoRI sites of pWPXL lentiviral vector (gift from D. Bach and D. Trono from EPH, Lausanne). Lentiviruses were produced and titrated as described [33]. C2C12 myoblasts were also transduced as described [33].

### Polyethylene glycol-mediated (PEG) cell fusion assay

This assay was adapted from Legros et al. [28]. PEG was purchased from BDH and DMEM from Gibco. Twenty-four hours after transduction of C2C12 cells stably expressing mtGFP and mtRFP, cells were washed with sterile PBS, trypsinized and counted; 1.8.10^5^ C2C12 mtGFP and 1.8.10^5^ C2C12 mtDsRed were coseeded on 25-mm coverslips in 6-well plates. Forty-eight hours after transduction, cells were washed three times with DMEM and then incubated during 30”–40” with 500 µl of PEG:DMEM (1:1 weight:volume). Cells were then washed gently three times with growth medium plus cycloheximide (100 µg/ml) and incubated with this medium. Four hours after of 30”–40” incubation with PEG, cells were fixed (PFA 4% in PBS) and mounted at room temperature.

Polykaryons from cell fusion experiments were counted (75–100 polykaryons per independent experiment) using a Nikon E1000 fluorescence microscope (FITC filter 465–490 nm excitation for mtGFP and G2A filter 510–560 nm for mtRFP) and were classified on the basis of their level of colocalization. Polykaryons classified as “High” contained at least 50% of their green and red mitochondrial matrix-targeted proteins mixed due to mitochondrial fusion (yellow mitochondria). Polykaryons with lower percentages were considered “Low”. Single stack representative images shown were taken with Leica laser scanning confocal microscope SP2 (laser emission 488 nm for mtGFP and 561 nm for mtRFP) using a 63X oil immersion objective (1.4) at room temperature. Images were magnified 2–3 times with Leica Lite ® software.

### Statistical analysis

Two-tailed *t* student test was performed in all experiments. *, # show statistical significance at distinct p values under 0.05.

### Supplemental Material

Figure S1 shows gene reporter experiments performed with a 2 Kb fragment of Mfn2 promoter and its mutated version without a functional Box 2. Figure S2 shows the quantification analysis of Porin induction by PGC-1β in total lysates of C2C12 differentiated muscle cells. Figure S3 shows the effect of PGC-1β overexpression on the abundance of different mitochondrial proteins expressed relative to Porin. Figure S4 shows representative images from different mitochondrial morphologies observed in Mfn2 mouse embryonic fibroblasts.

## Supporting Information

Figure S1 Box 2 mutation in Mfn2 promoter blocks PGC-1β-dependent ERRα coactivation. 10T1/2 cells were transfected with a 2-Kb (−1982/+45) fragment of Mfn2 promoter (white bars) or its mutated version (Mfn2 promoter Box2 Mut, black bars) together with an irrelevant vector (Basal), 75 ng of PGC-1β expression vector, ERRα or with PGC-1β+ERRα. Graphs display mean±SEM luciferase related to renilla activity values of 3 independent transfection experiments performed in triplicate. *Statistical difference compared to basal group, p<0.05. #Statistical difference compared to wild-type Mfn2 promoter, p<0.05.
(0.52 MB TIF)

Figure S2 Porin expression is increased ∼78% in C2C12 myotubes transduced with PGC-1β adenovirus. Total lysates were obtained from non-transduced (NT), LacZ- or PGC-1β-transduced C2C12 myotubes at MOI 100 during 48 h and analyzed by Western blot with specific antibodies raised against Porin. Graphs show mean±SEM of Porin densitometric quantification levels related to LacZ Porin values from 4 independent differentiation and transduction experiments. *Statistical difference, p = 0.01.
(0.43 MB TIF)

Figure S3 Normalization of mitochondrial dynamics components and ETC subunits protein levels induced by PGC-1β by Porin levels. Quantification analysis of protein levels of mitochondrial dynamics components and the ETC subunits from complexes I to V detected by Western blot and related to porin levels. Mean±SEM of Non- (white bars) , LacZ- (grey bars) or PGC-1β- (black bars) transduced C2C12 myotubes from n = 4 independent differentiation and transduction (MOI 100) experiments. *, statistical difference compared to LacZ transduction, p<0.05.
(0.74 MB TIF)

Figure S4 Representative images from distinct transfected Mfn2 −/− MEFs classified on the basis of mitochondrial morphology. Mitochondria were labelled with anti-Cox1 antibody detected with a secondary antibody conjugated to Alexa 594. Scale bar, 10 µm.
(5.83 MB TIF)
