# Supplementary figures and images for: Mitochondrial Fusion Is Increased by the Nuclear Coactivator PGC-1β
Source: PLoS One. 2008 Oct 31;3(10):e3613. doi: 10.1371/journal.pone.0003613 (PMC2570954; doi:10.1371/journal.pone.0003613)

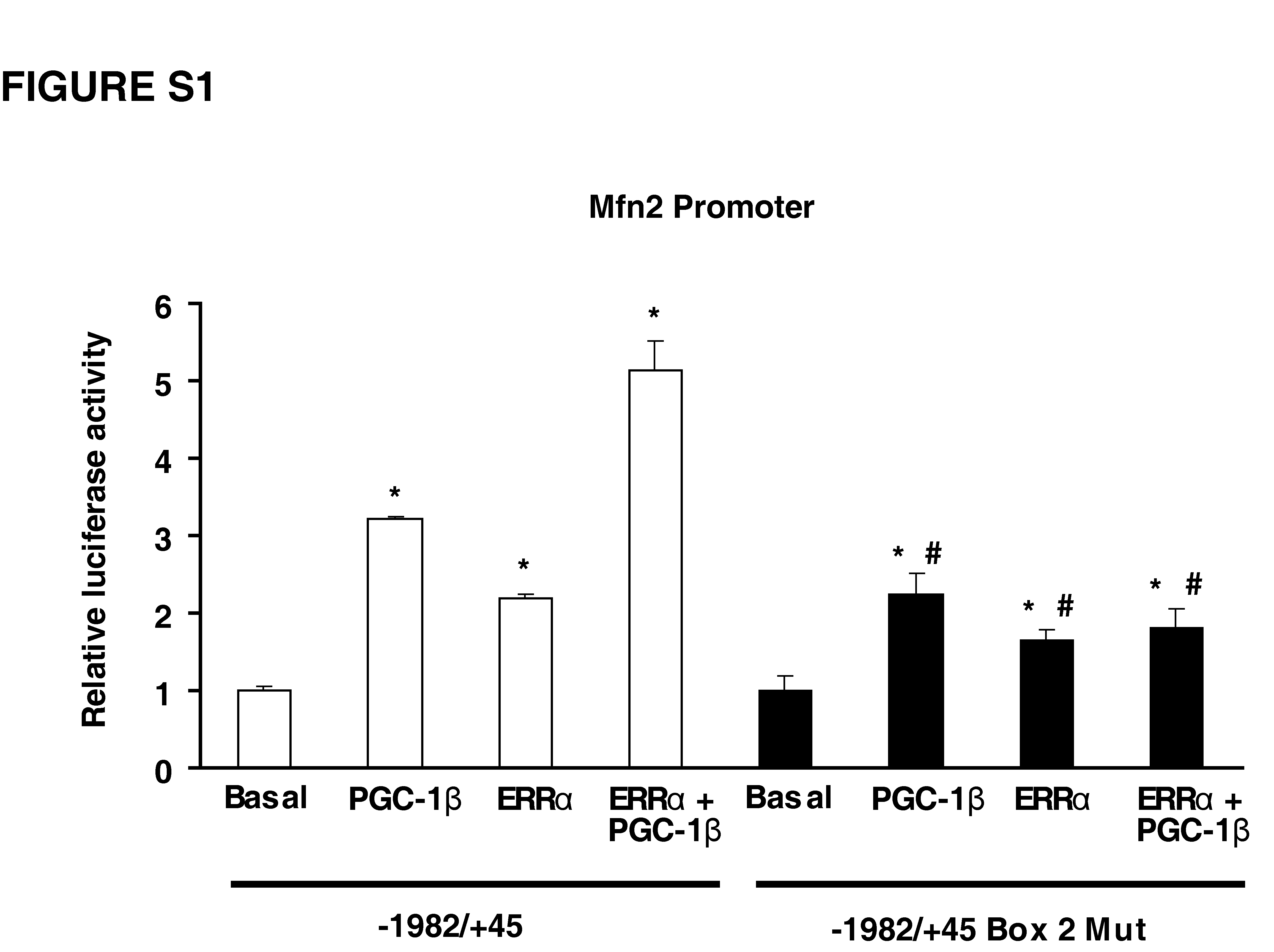

Supplement: Figure S1 — Box 2 mutation in Mfn2 promoter blocks PGC-1β-dependent ERRα coactivation. 10T1/2 cells were transfected with a 2-Kb (−1982/+45) fragment of Mfn2 promoter (white bars) or its mutated version (Mfn2 promoter Box2 Mut, black bars) together with an irrelevant vector (Basal), 75 ng of PGC-1β expression vector, ERRα or with PGC-1β+ERRα. Graphs display mean±SEM luciferase related to renilla activity values of 3 independent transfection experiments performed in triplicate. *Statistical difference compared to basal group, p<0.05. #Statistical difference compared to wild-type Mfn2 promoter, p<0.05. (0.52 MB TIF) [file pone.0003613.s001.tif]

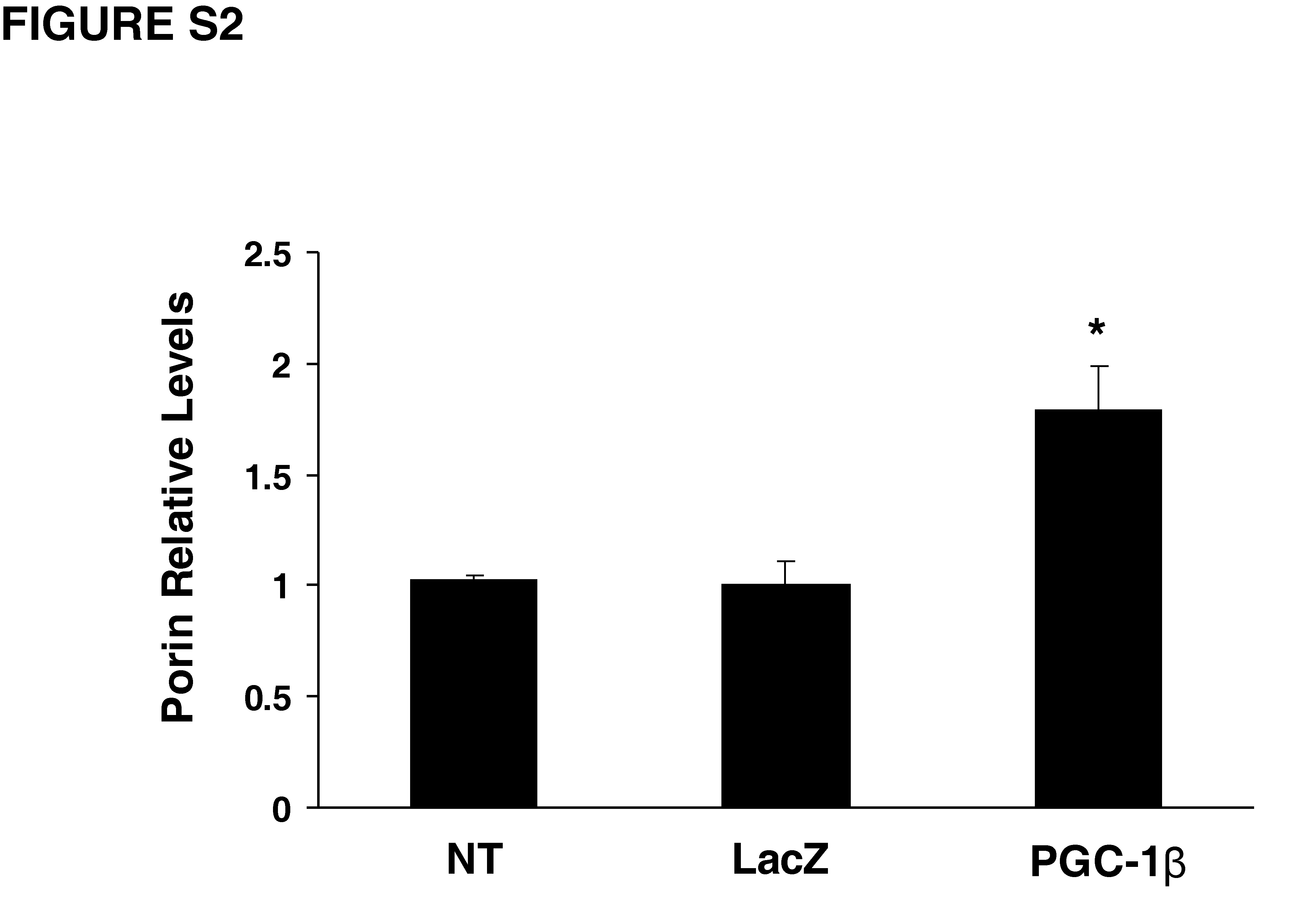

Supplement: Figure S2 — Porin expression is increased ∼78% in C2C12 myotubes transduced with PGC-1β adenovirus. Total lysates were obtained from non-transduced (NT), LacZ- or PGC-1β-transduced C2C12 myotubes at MOI 100 during 48 h and analyzed by Western blot with specific antibodies raised against Porin. Graphs show mean±SEM of Porin densitometric quantification levels related to LacZ Porin values from 4 independent differentiation and transduction experiments. *Statistical difference, p = 0.01. (0.43 MB TIF) [file pone.0003613.s002.tif]

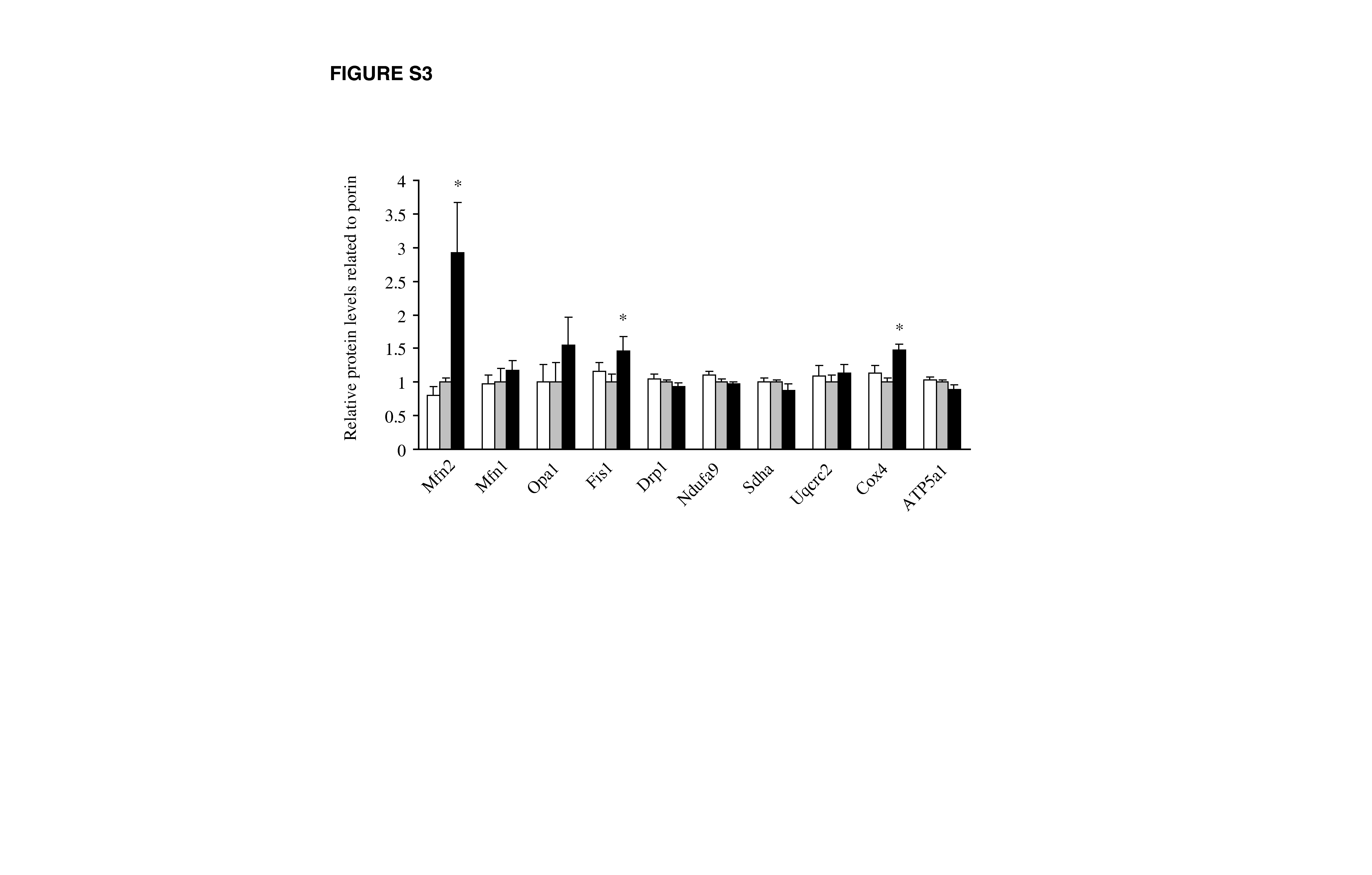

Supplement: Figure S3 — Normalization of mitochondrial dynamics components and ETC subunits protein levels induced by PGC-1β by Porin levels. Quantification analysis of protein levels of mitochondrial dynamics components and the ETC subunits from complexes I to V detected by Western blot and related to porin levels. Mean±SEM of Non- (white bars) , LacZ- (grey bars) or PGC-1β- (black bars) transduced C2C12 myotubes from n = 4 independent differentiation and transduction (MOI 100) experiments. *, statistical difference compared to LacZ transduction, p<0.05. (0.74 MB TIF) [file pone.0003613.s003.tif]

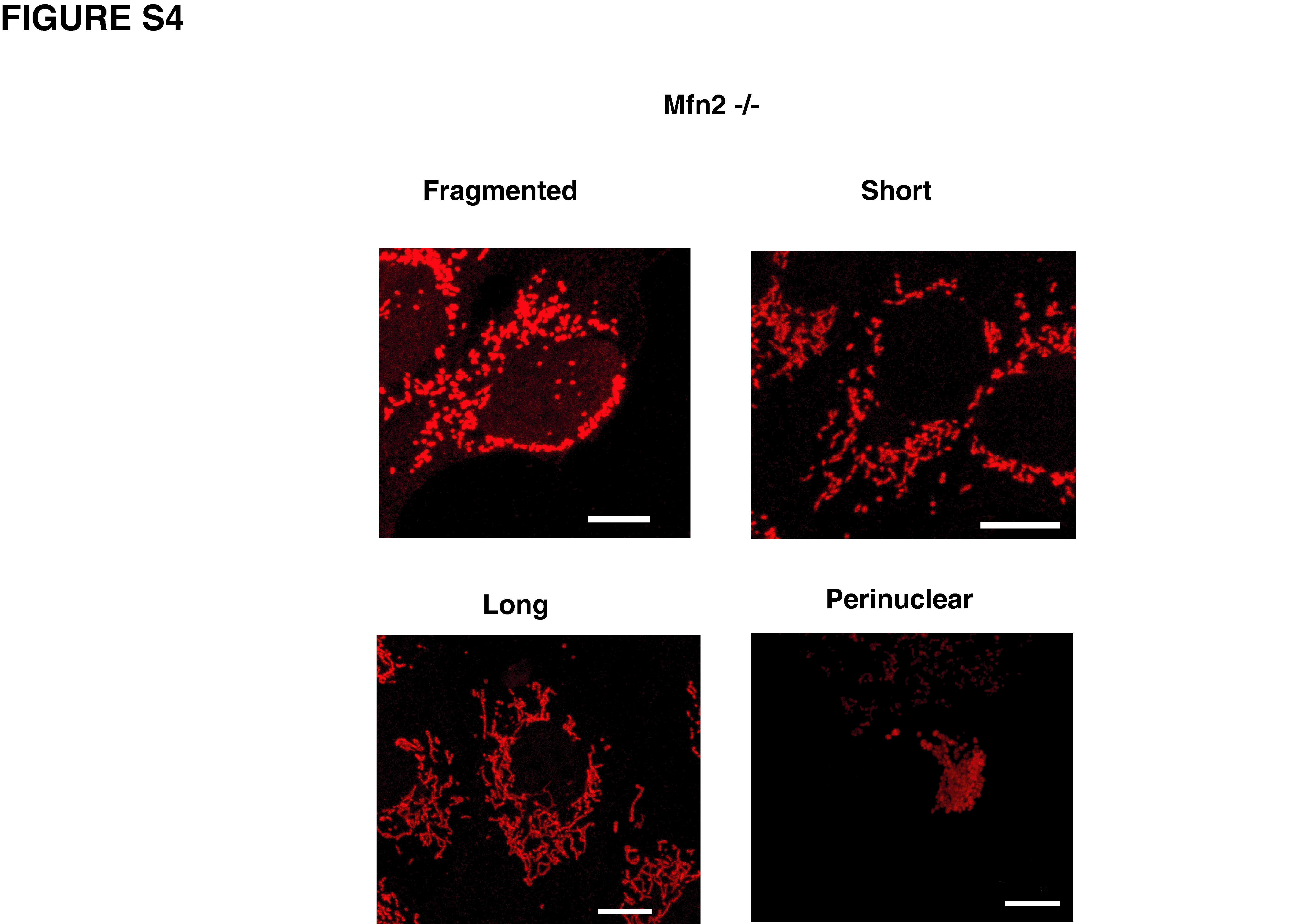

Supplement: Figure S4 — Representative images from distinct transfected Mfn2 −/− MEFs classified on the basis of mitochondrial morphology. Mitochondria were labelled with anti-Cox1 antibody detected with a secondary antibody conjugated to Alexa 594. Scale bar, 10 µm. (5.83 MB TIF) [file pone.0003613.s004.tif]
